# Motivators for Physical Activity among Ambulatory Nursing Home Older Residents

**DOI:** 10.1155/2014/329397

**Published:** 2014-06-25

**Authors:** Yuh-Min Chen, Yueh-Ping Li

**Affiliations:** ^1^School of Nursing, China Medical University, No. 91, Hsueh Shih Road, Taichung 40402, Taiwan; ^2^Department of Nursing, China Medical University Hospital, Taichung 40402, Taiwan; ^3^Department of Nursing, College of Medicine and Life Science, Chung Hwa University of Medical Technology, Tainan 71703, Taiwan

## Abstract

The purpose of this study was to explore self-identified motivators for regular physical activity among ambulatory nursing home older residents. A qualitative exploratory design was adopted. Purposive sampling was performed to recruit 18 older residents from two nursing homes in Taiwan. The interview transcripts were analyzed by qualitative content analysis. Five motivators of physical activity emerged from the result of analysis: eagerness for returning home, fear of becoming totally dependent, improving mood state, filling empty time, and previously cultivated habit. Research on physical activity from the perspectives of nursing home older residents has been limited. An empirically grounded understanding from this study could provide clues for promoting and supporting lifelong engagement in physical activity among older residents. The motivators reported in this study should be considered when designing physical activity programs. These motivators can be used to encourage, guide, and provide feedback to support older residents in maintaining physical activity.

## 1. Introduction

Age-related changes result in decreasing physiological reserve which leads to vulnerability to diseases and functional impairments. Remaining active throughout later life can have a positive effect on the aging process. Regular physical activity enhances physiological stability as well as functional ability and minimizes the debilitating effects of disuse [1–4]. Physical activity particularly offers an excellent way for mobility-impaired older adults to prevent further disabilities and improve their overall health [5, 6]. On the other hand, sedentary living is a main health risk for older adults. Low levels of physical activity lead to deconditioning and are associated with increasing risks of morbidity and mortality [7–9].

Although physical activity is a vital part of healthy aging, physical activity levels often decrease with age [2, 10]. This is even more evident for nursing home residents. Weeks et al. [11] found that physical activity levels of nursing home residents were lower than community-dwelling older adults. MacRae et al. [12] reported that the majority of ambulatory nursing home residents were inactive and automatically limited their physical activity. Similarly, the study by Lee et al. [13] also found that physical activity levels of nursing home residents were relatively low. For nursing home older residents, abilities and environmental resources for engaging in health behaviors are more restricted than community-dwelling older adults. Therefore, these residents are more likely to lead a sedentary lifestyle. However, previous research has indicated that older residents in nursing homes can still have very positive experiences and gain beneficial outcomes with exercise participation [14–16].

Even though physical inactivity is a pervasive problem among nursing home residents, there are a part of residents that still engage in regular physical activity. What motivates them to keep physically active? Early studies on physical activity have mainly focused on community-dwelling older adults. Additionally, the majority of research on physical activity and older adults used quantitative design focusing on exploring predictors or determinants of physical activity. Few extant studies applied qualitative design to explore experiences related to physical activity of older adults in long-term care settings. Weeks et al. [11] adopted qualitative design to identify factors influencing physical activity participation among older adults in the community and in a nursing home. They found that past experiences, life transitions, and future concerns affected older adults' participation in physical activity. Another qualitative study by Phillips and Flesner [17] using focus group and the Health Promotion Model as a guiding framework investigated individual and situational influencing factors of physical activity of older adults in residential-care/assisted-living communities. The result indicated 6 themes related to physical activity: staying active, past physical activity experiences, value of physical activity, barriers to physical activity, strategies to facilitate physical activity, and support needs to promote physical activity. These two studies provide more detailed knowledge regarding perspectives of physical activity of older adults in long-term care facilities. However, they did not specifically focus on motivators and older adults in the nursing homes. Additionally, the exploring focus of Phillips and Flesner's [17] study was limited to variables in the Health Promotion Model. There is limited in-depth information about motivators for physical activity of nursing home older residents. More studies are needed to advance our understanding of this phenomenon. Therefore, the aim of this study was to explore self-identified motivators for regular physical activity among ambulatory nursing home older residents.

Given the dearth of research on physical activity motivators among nursing home residents, a qualitative approach was used to explore residents' experiences. Qualitative research is invaluable because it enables nursing home staff to understand nursing home residents' perspectives and thus helps address their needs. The findings of this study could expand nursing home staff's knowledge of how to support older residents to continue engaging in regular physical activity and to create a supportive environment for active aging.

## 2. Methods

A qualitative exploratory design was adopted to collect data from ambulatory nursing home older residents about their perceived motivators for physical activity.

### 2.1. Participants

Purposive sampling was used to recruit participants according to the following inclusion criteria: (1) being 65 years of age or older, (2) being capable of ambulating independently with or without the use of an assistive device, (3) residing in the nursing home for at least six months, (4) engaging in any physical activities for a minimum of 30 minutes on 5 days each week, and (5) being able to communicate. The sample size was determined by data saturation. That is, no new information was discovered and data redundancy was achieved [18]. However, to ensure that saturation was really achieved, two more additional participants were interviewed. At last, the purposive sample included 18 older residents. They were recruited from two for-profit nursing homes in Taiwan. Participants were aged between 68 and 93, with a mean of 80.61 years (SD = 7.28). The majority of the participants were male (61.1%) (Table 1).

### 2.2. Data Collection

Ethical approval for the study was obtained from the Research Ethics Committee of China Medical University and Hospital (number CMU-REC-100-011). In the nursing home, the manager and nurses identified eligible residents and introduced the researcher to the potential participants to help establish rapport. Then, the researcher explained the research purpose and the interviewing process to potential participants. After the informed consent was obtained, an interview was conducted with each participant by the first researcher. The interviews were held in the participants' own rooms or the garden, according to participants' preferences. For building relationships, the interviews typically started with a warm-up conversation. The researcher introduced herself and chatted with the participant. Then the interview began by asking the participant to talk about what motivates them to engage in regular physical activity. Probing questions were used to elicit more detailed information from the participants. With participants' permission and further reassurances of confidentiality, interviews were audiotaped. Each interview lasted approximately 30–40 minutes.

### 2.3. Data Analysis

After each audiotaped interview was transcribed verbatim, qualitative content analysis was used for data analysis [19]. Initially, to grasp an overall understanding, all transcribed texts were read several times. Then, meaningful statements that expressed a complete idea and directly related to the study object were extracted and their underlying meanings were coded. Coding categories were progressively refined until all data were coded into exclusive categories.

### 2.4. Rigor

Trustworthiness of the study was enhanced using the criteria proposed by Lincoln and Guba [20]: credibility, transferability, dependability, and confirmability. A number of approaches were used. Credibility was ensured by peer debriefing. The analysis was discussed with an expert in qualitative research as well as long-term care. To establish transferability, thick descriptions and direct quotations were provided to enable potential appliers to evaluate the applicability of the findings to others in similar situations. Dependability was strengthened by inquiry audit whereby the selected transcripts were reviewed by a peer for consensus regarding emerging categories. To ensure confirmability, the audio tapes, transcribed interview texts, and data analysis results were all preserved to establish an audit trail.

## 3. Results

Five motivators of physical activity emerged from the result of analysis: eagerness for returning home, fear of becoming totally dependent, improving mood state, filling empty time, and previously cultivated habits.

### 3.1. Eagerness for Returning Home

For six participants, returning to their own home functioned as an effective motivator. The eagerness of returning home drove them to engage in regular physical activity. They believed that if their physical functions improve, the probability of getting back to their homes would also increase. They expressed the following:

*“I cannot walk steadily and need a wheelchair. I participate in rehabilitation activities to improve the muscle power of my legs, so as to stand up again. … I hope I can get back to my own home as soon as possible. I do not want to continue to live here. Here is not my home. I want to get back to my own home and be with my family.”*

*“I participate in the rehabilitation program every day and practice walking by using a walker several times a day. The reason pushing me to do so is the hope of getting back to my own home. … My husband had a stroke and is cared by an attendant at home. I miss him very much and want to go home to stay with him. … However, my daughter said it is difficult and dangerous to take care of both of us at home. So, I think if I do some exercise to help my legs become stronger, when my daughter sees the improvement of my body function, she might consider letting me go home and I would not have to continue to live in this institution. Therefore, I practice walking as frequently as I can to strengthen the muscle powers of my legs.”*

### 3.2. Fear of Becoming Totally Dependent

Ten participants identified functional independence and mobility as the main reasons for engaging in regular physical activity. They were afraid that the loss of mobility would cause them to become totally dependent and therefore have to be reliant upon others for assistance with daily activities. Participants stated the following:

*“I use a wheelchair to practice walking every day. By doing this, I can strengthen the muscle power and endurance of my legs. Although the improvement seems unapparent, I believe that by keeping walking every day, I can at least prevent degeneration. If my legs continue to degenerate, I will not be able to move around by myself. I will need to depend upon the staff for almost everything. The staff members here are always busy and cannot respond to our requests promptly. If I need them to help me with everything, I will always have to wait for a long time. … This will be too pitiful.”*

*“One important thing I do every day is to participate in rehabilitation activities. I hope that this will help me regain some functions of my left hand and leg and prevent degeneration of my right hand and leg. I do not expect that my body function will return to a normal state. But, at least I hope I can move more freely. Then I will be able to do most of things by myself without bothering other persons for help. For me, being able to stand up, go to a toilet by myself, walk freely…, these are all so important and give me confidence. Becoming totally dependent is a very terrible thing and means the loss of dignity. I do not want to become so miserable. … Every time when I think about these situations, I work harder in rehabilitation activities.”*

### 3.3. Improving Mood State

For participants, admission to nursing homes led to not only a change in physical location of an original living space, but also separation from family members. Additionally, the public and cohabitation nature of nursing home life was not easy for participants to accept. Their emotions were influenced by these living situations. Six participants mentioned avoiding depressed and bad moods as well as enhancing positive moods as motivators for physical activity. They stated the following:

*“The nursing home is not a good place to spend the rest time of my life. However, I have no choice. … I miss my family very much. I feel sad every time when I think about my family. … Doing some physical activities can help me have a better mood. I take a walk and perform gentle calisthenics every morning after breakfast. Keeping regular exercise is good for me because in this way I do not get depressed. I often feel better when I exercise.”*

*“The hygienic habits and some behaviors of several residents here often make me feel uncomfortable. Additionally, one of my roommates is very dominant and often argues with other residents. It is not easy to have a good mood all the time. The garden has the fresh air and some enjoyable scenery. Taking a walk outside can help me get rid of a bad mood. It helps me stop thinking about anything unhappy. … It helps me have a more cheerful mood.”*

### 3.4. Filling Empty Time

Because of the fixed schedule of daily activities and lack of choices and access to various activities, eight participants complained about long days and a monotonous lifestyle in the nursing home. Therefore, they engaged in regular physical activities to enrich their lives in the nursing home.

*“Nursing home life is nothing but a fixed schedule of daily routine. There is nothing interesting to do here, so you have to find something to do by yourself to kill time. The thing which I do is to take a walk every day.”*

*“Life here is very boring. In addition to meals and snack time, there is nothing else to do. I even have very few people whom I can talk to … I do not want to spend all my time in the room. Because of boredom, I go for a walk twice a day to kill time.”*

### 3.5. Previously Cultivated Habits

The importance of a daily physical activity routine was also a prominent factor among participants. Seven participants attributed their maintenance of a specific physical activity to a previously cultivated habit. They strongly emphasized a consistent daily physical activity routine. These participants reported a daily physical activity routine which included walking, stretching calisthenics, and arm swinging.

*“I go for a walk every day and never miss a day. This has become a habit. It's a part of daily activity.”*

*“I used to take a walk every day and perform gentle calisthenics from several years ago. These activities are important daily routines.”*

*“I take a walk for about 20 minutes and doing arm swinging Tai Chi every day. This habit has been maintained for more than 4 years. I never stop doing so, even when I moved here two years ago. I think that I will maintain this activity as a habit until one day I cannot move myself any longer. These activities can relieve my constipation and are good for my body.”*

## 4. Discussion

The result of data analysis revealed five major motivators of regular physical activity, including eagerness for returning home, fear of becoming totally dependent, improving mood state, filling empty time, and previously cultivated habits. Using qualitative interviews, participants were able to openly express their experiences and perspectives. It is thus helpful for gaining useful insight into older residents' concerns. Moreover, motivation is influenced by cultural factors [21]. Past qualitative studies on physical activity were conducted in western countries. The findings of this study contribute not only to the scant knowledge of motivators for physical activity among nursing home residents, but also to the provision of culturally sensitive care.

Physical activity improves residents' physical capacities and overall health, which in turn has a positive impact on their quality of life. Motivation drives a person to greatness [22]. It is suggested that the motivators reported in this study should be considered when designing physical activity programs. Nurses should be proactive in motivating older residents to adopt an active lifestyle. These motivators can be used to encourage, guide, and provide feedback to support older residents in maintaining physical activity levels.

For almost all of people, the meaning of home encompasses a broad realm of memory and feelings. Home is the place where one lives and is associated with special affective relationships [23], continuity through time [24], and deep existential meaning [25]. Relocation to the nursing home forced participants to leave their homes and often resulted in the feeling of broken connections with family members. Moreover, in traditional Chinese culture, older parents are supposed to be cared for by their adult children at home. It is filial obligation and cultural expectation. Therefore, it is understandable how an eagerness for returning home motivated participants to engage in regular physical activity, especially in rehabilitative efforts. Similarly, the qualitative study by H. H. Tsai and Y. F. Tsai [26] revealed that older Taiwanese nursing home residents viewed the nursing home as “a temporary home to nurture health” instead of a “real home.” They believed that they would go home if their health condition improved. Unfortunately, due to the limitation of home care resources, some older residents may still not be able to return to their homes, even though their physical functions improve. However, family members are always in a unique position to influence and afford valuable support to older residents. Family visitation and involvement are important for older residents. Nursing home staff should encourage family members to regularly interact with older residents to provide them emotional strength to continue with physical activity. Furthermore, family members can be considered as partners in care [27]. Nursing home staff can develop ways to enhance family involvement in the nursing home care to provide ongoing support for older residents.

Mobility and functional level are the major abilities necessary for an independent life. Being mobile also relates to self-worth and quality of life [28, 29]. For older residents, preventing degeneration and maintaining residual physical functions to allow them to perform basic daily activities mean that they still have some control over their life. On the other side, becoming totally dependent on nursing home staff can lead to emotional suffering, such as sense of helplessness and loss of confidence. Therefore, fears of becoming totally dependent act as a driving force in participants' engagement in physical activity. Similarly, Weeks et al. [11] reported that preventing further health decline is a key motivator for physical activity participation for older adults. As Prata and Scheicher [30] indicate, loss of independence is one of older adults' major concerns. Experiencing control over life can increase life satisfaction [31] and quality of life [32]. Knowing what older adults see as important is a key part of compassionate relationship-centred care [33]. Nursing home staff should have awareness that promoting physical functions is important for maintaining an older resident's well-being, both physically and psychologically. It is therefore suggested that nursing home staff should incorporate appropriate interventions into routine care to enable older residents to prevent progressive disability and achieve their highest possible level of functional independence.

Nursing home life is highly collective [33]. Participants had no choices but to live with a various mix of other residents who were heterogeneous in terms of personal characteristics and lifestyles. Their emotions were easily affected by uncomfortable feelings aroused in everyday interaction with other residents. To adjust the difficulties arising from communal living, participants engaged in physical activities to improve their mood. Their response was partially influenced by the Chinese sociocultural value which stresses that avoiding conflicts in social relationships and maintaining interpersonal harmony are helpful for having peaceful life. Therefore, instead of arguing with others, participants chose their own ways to improve mood. Moreover, this finding supports that participation in meaningful activities is important to promote not only physical health but also psychosocial health [34, 35]. In addition to supporting residents' original physical activities, nursing home staff should develop feasible and meaningful programs to enhance social adaptations and positive interactions among residents, whereby physical activity can also be further strengthened.

Due to a lack of various and interesting activity programs in the nursing homes, some participants practiced physical activity to fill empty time and relieve their sense of boredom. Prior studies also point out that institutionalized residents often lead a monotonous and tedious lifestyle and restrictions on activity choices [31, 33, 36, 37]. Lack of opportunities for enriching activities can cause low levels of activity engagement [17]. Although the monotonous characteristics of nursing homes function as motivators for physical activity, making physical activity fun and not necessarily just for killing time would be more enjoyable and encourage residents to participate more in activities. Kane [38] proposes meaningful activity to be one of the eleven domains of good quality of life for those residing in long-term care facilities. Nolan et al. [39] also assert that to create an enriched environment, older adults need to have opportunities to engage in purposeful activity and enjoy meaningful activity. The nursing home staff should first discover what activities the residents desire and enjoy. Moreover, availability and adequacy of environmental resources are also vital factors [17]. Thus, the staff members have to offer opportunities as well as resources and consider environmental changes to become more vibrant to support residents to pursue individual preferences and meaningful activities. In so doing, the lives of residents will be enriched when their desires are met [40].

Several participants mentioned the importance of keeping a daily physical activity routine. The habits of physical activity established in previous life stages tend to persist over time [34]. The result of the present study is consistent with previously reported findings that prior established exercise habits play an influential role in current exercise behavior [11, 37, 41, 42]. Interventions would be more productive if they are built on prior physical activity habits and preferences. Habit strength can be augmented by repetitive practice of behavior [34]. As a result, the residents' likelihood of maintaining physical activities would possibly increase.

Because of the nonrepresentative sampling, results from this study should be interpreted with caution. The participants of this study were recruited from two nursing homes. Although the sample size was appropriate for qualitative research, a greater representation of older residents might strengthen the collection of more saturated data. A more comprehensive understanding of motivators of physical activity would further advance our understanding toward this topic. Additionally, motivators might change over time. Future research can track older residents' motivators for physical activity over time. Longitudinal research will add valuable information to the existing knowledge.

## 5. Conclusion

Research on motivators of physical activity from the perspectives of nursing home older residents has been limited. The results provide nursing home staff with a unique view of what motivates nursing home residents to engage in regular physical activities. The ultimate goal of caring for older residents is to help them live a better quality life by maximizing their independence and health. It is expected that an empirically grounded understanding from this study could provide clues for promoting and supporting lifelong engagement in physical activity among nursing home residents.

## Figures and Tables

**Table 1:** Characteristics of research participants (*N* = 18).

Variables	*N*	%	Mean	SD
Age			80.61	7.28
65–74	3	16.7		
75–84	10	55.5		
≥85	5	27.8		
Gender				
Male	11	61.1		
Female	7	38.9		
Marital status				
Married	3	16.7		
Widowed	14	77.8		
Divorced	1	5.5		
Comorbidities			2.28	0.90
Hypertension	14	77.8		
Osteoarthritis	5	27.7		
Diabetes	5	27.7		
BPH	4	22.2		
CVA	4	22.2		
Cataract	3	16.7		
CHF	2	11.1		
Liver cirrhosis	1	5.5		
CKD	1	5.5		
CAD	1	5.5		
Gout	1	5.5		
Functional status (measured by Barthel index)			80.0	5.94
90	2	11.1		
85	4	22.2		
80	6	33.4		
75	4	22.2		
70	2	11.1		
Reasons for nursing home admission				
Declining health, family unable to provide care	5	27.7		
Declining health, lack of home care resources	13	72.3		
